# Psychobiotics Ameliorate Depression and Anxiety Status in Surgical Oncology Patients: Results from the *ProDeCa* Study

**DOI:** 10.3390/nu17050857

**Published:** 2025-02-28

**Authors:** Georgios Tzikos, Eleni Chamalidou, Dimitra Christopoulou, Aikaterini Apostolopoulou, Sofia Gkarmiri, Marianthi Pertsikapa, Alexandra-Eleftheria Menni, Ioannis M. Theodorou, George Stavrou, Nektaria-Dimitra Doutsini, Anne D. Shrewsbury, Theodosios Papavramidis, Joulia K. Tsetis, Helen Theodorou, Anastasia Konsta, Katerina Kotzampassi

**Affiliations:** 1 Department of Surgery, Aristotle University of Thessaloniki, 54636 Thessaloniki, Greece; gtziko@auth.gr (G.T.); dimitraxristopoulou9@gmail.com (D.C.); alexmenn@auth.gr (A.-E.M.); nektariadout@gmail.com (N.-D.D.); a_shrewsbury@yahoo.com (A.D.S.); tpapavra@auth.gr (T.P.); 2 Outpatient Surgical Oncology Unit, Chemotherapy Department, AHEPA University Hospital, Aristotle University of Thessaloniki, 54636 Thessaloniki, Greece; eleniham@gmail.com; 3 Department of Emergency Medicine, AHEPA University Hospital, Aristotle University of Thessaloniki, 54636 Thessaloniki, Greece; aapostod@auth.gr (A.A.); sgkarmi@auth.gr (S.G.); marianpertsis@gmail.com (M.P.); 4 Hellenic Institute for the Study of Sepsis (HISS), 11528 Athens, Greece; itheodorou@med.uoa.gr; 5 Department of Surgery, 417 NIMTS (Army Share Fund Hospital), 11521 Athens, Greece; stavgd@gmail.com; 6 Uni-Pharma S.A., 14564 Athens, Greece; jtsetis@uni-pharma.gr; 7 Department of Sociology, School of Social Sciences, University of Crete, 74100 Rethymno, Greece; psy5057@psy.soc.uoc.gr; 8 First Department of Psychiatry, “Papageorgiou” General Hospital of Thessaloniki, Aristotle University of Thessaloniki, 54124 Thessaloniki, Greece; konstaa@auth.gr

**Keywords:** psychobiotics, depression, anxiety, insomnia, perceived stress, surgical oncology, chemotherapy, gastrointestinal cancer

## Abstract

**Background**: Psychological disorders are prevalent in patients having undergone gastrointestinal cancer surgery, and their emotional status may further deteriorate during subsequent chemotherapy. Psychobiotics are specific probiotics that have the unique characteristics of producing neuroactive substances that are thought to act on the brain–gut axis. The aim of the present study was to evaluate the benefits of a psychobiotic formula on depression and anxiety status, as well as on perceived stress, versus a placebo in patients on a chemotherapy course following gastrointestinal surgery for cancer. **Patients**: The enrolled patients, allocated to the psychobiotic and placebo groups, were assessed by means of these psychometric tests: Beck’s Depression Inventory and the Hamilton Depression Rating 17-item Scale for depression; the General Anxiety Disorder-7 for anxiety; and the Perceived Stress Scale-14 Item for perceived stress at three time-points: upon allocation [T1], after one month of treatment [T2], and two months thereafter [T3]. **Results**: In total, 266 patients were included. One month of psychobiotic treatment improved [i] depression status by 60.4% [48 depressed patients at T1, reduced to 16 at T3]; [ii] anxiety by 57.0% [72 patients at T1, 26 at T3]; and [iii] stress by 60.4% [42 at T1, 14 at T3]. The placebo-treated patients experienced a deterioration in all parameters studied, i.e., depression increased by 62.9%, anxiety by 39.7%, and stress by 142.5%. **Conclusions**: Based on these findings, it can be recognized that psychobiotic treatment has great potential for every patient at risk of suffering from depression, anxiety, or stress during the course of surgery/chemotherapy for gastrointestinal cancer.

## 1. Introduction

Cancer diagnosis is a stressful situation that could be life-changing. Diagnosing cancer initially provokes feelings of anger, “why me”, and anxiety, “what to do, now and from here on”. Then, emotional turmoil follows, such as worry, sadness, nervousness, hopelessness, and loss of interest in daily activities [1,2,3]. Although surgery, and [neo]-adjuvant chemo-radiotherapy—of any type—are now considered, in general, as effective treatments, many other co-factors are involved: [a] the primary disease site; [b] the cancer stage and prognosis; [c] the treatment modalities; [d] the fear of disease progression and recurrence; and [e] malnutrition and/or sarcopenia. In the case of life-threatening cancers, along with loss of the autonomy, the sense of uncertainty, acute loss of perspective on life, sense of isolation, poor quality of life, fear of pain and adverse outcomes, and, particularly, the fear of death are also involved [2,3,4,5,6].

To those general negative emotional feelings, some cancer types add their discrete morbidity and some specific psychological issues: anorectal surgeries may involve permanent or temporary stoma, diarrhea and incontinence [7,8,9], gastric surgeries, reflux, dysphagia, eating restrictions, dyspnea, heartburn, and malnutrition [10]. Head and neck cancers commonly involve tracheostomy and, therefore, loss of voice and communication, and loss of taste and smell, as well as the inability to chew and enjoy eating and drinking [11,12,13]. Finally, complications related to adjuvant therapy may include nausea, vomiting, diarrhea [14,15,16], hair loss, fatigue, insomnia, and cognitive impairment after chemotherapy [17,18]. Long treatment periods, repeated hospitalizations due the side-effects of chemotherapy, and prolonged unemployment, in combination with low income [19], a low educational level, and parameters of gender, marital status, and religion, can all affect the psyche of cancer patients [14].

In the cases where the emotional burden exceeds a patient’s ability to cope, specific psychiatric syndromes, such as prolonged stress, anxiety, and depressive disorders, may arise and are, in fact, relatively common [20]. Basak et al. [21] examined the prediction of anxiety and depression following abdominal surgery for cancer and revealed a rate of 31% to 56%, respectively, at the time of hospital discharge. Other studies have shown similar results of 30% to 40% [4,22,23]. When the Hospital Anxiety and Depression Scale (HADS) score was applied to a total of 200 gastric cancer patients scheduled for extended gastrectomies, a prevalence of 33.5% vs. 10.0% [*p* < 0.001], a HADS-anxiety score of 7.4 ± 3.8 vs. 4.7 ± 2.8, [*p* < 0.001], and a HADS-depression score of 6.9 ± 3.5 vs. 4.2 ± 2.6, [*p* < 0.001] in relation to healthy controls were reported [10]. Among all the cancer sufferers, the gastric cancer patients had the highest rate of mixed anxiety/depression symptoms [24]. Similarly, in anorectal cancer patients, depression and anxiety was positively correlated with persistent diarrhea and other gastrointestinal symptoms [7], while the fear of incontinence [8] or of odor from a stoma interfered with social functioning, thus increasing their psychological distress [9]. According to Purkayastha et al. [25], the prevalence rate for depression is three-fold higher in the cancer population in relation to the healthy population, and those at risk of suicide increased 2.5-fold [26]. Finally, in a study of 263 cancer patients [19] who were undergoing chemotherapy, fatigue, insomnia, anxiety, and depression were found to be positively correlated to each other and negatively correlated with the quality of life [*p* < 0.001], with the highest impact relating to depression.

Among 106 rectal cancer patients receiving neoadjuvant chemoradiation, 42% experienced acute toxicities and 24.5% late toxicities [27]. The data also suggest that cancer itself, along with chemotherapy and radiotherapy treatments, is implicated in persisting modifications of inflammatory setpoints through the epigenetic effects of genes related to inflammatory signaling pathways [11], leading to the activation of the neuroimmune response [28,29] through the hypothalamic–pituitary–adrenal (HPA) axis, and, thus, is linked to increased stress and neuro-psychiatric conditions [28,30,31,32]. It is also well known that depression and anxiety are the result of the inappropriate production of serotonin, dopamine, norepinephrine, and other neurotransmitters that are critical for proper mood functioning [28,29,30,33]. On the other hand, the increased expression of free radicals and of the pro-inflammatory cytokines by the cancer and the interventions for its treatment affect the microbiome composition and diversity [33,34,35], which is well known to be involved in mood disorders [30,36] through the nervous, neuroendocrine, and immune systems [33,37,38,39].

Psychobiotics, a specified group of probiotics, the name of which stands for probiotics that support mental health benefits in patients suffering from psychiatric illness, have been one of the most discussed interventions since their introduction by Ted Dyan and John Cryan in 2013 [40,41]. They have the unique characteristics of producing and delivering neuroactive substances such as gamma-aminobutyric acid and serotonin, which act on the brain–gut axis [42,43], and they generally exert beneficial effects on stress, anxiety, depression, insomnia, and other psychopathological disorders [44,45,46] by modifying the disturbed microbiome abundance [41,47].

In the present prospective, double-blind, and randomized trial, we decided to evaluate a probiotic formulation containing bacteria with well-recognized “psychobiotic properties” relating to mood performance in patients on a course of chemotherapy following gastrointestinal surgery for cancer by means of standard psychometric assessment tools. Our primary target was to evaluate depression status changes pre- and post-treatment and two months thereafter; the secondary outcomes were to estimate changes in anxiety, perceived stress, and sleeping disorders at the same time-points versus placebo treatment.

## 2. Materials and Methods

This was a randomized, double-blind, and placebo-controlled trial performed on patients undergoing chemotherapy who had previously been operated on for gastrointestinal cancer. This study, abbreviated to ‘*ProDeCa*’ [PRObiotics for DEpression in CAncer] was conducted in the First Surgical Department of the AHEPA University Hospital of Thessaloniki, Greece, in collaboration with the Outpatient Surgical Oncology Unit, Chemotherapy Department, of the same institution, and started in November 2023.

### 2.1. Ethical Considerations

The study protocol was approved by the Ethics Committee of the AHEPA University Hospital of Thessaloniki, Greece (Scientific Council of the AHEPA Hospital, 313/4 July 2023), and was prospectively registered with ClinicalTrials.gov (NCT 06496438).

Written and oral informed consent was obtained from all eligible participants, and they were assured that they could withdraw from the study at any time.

### 2.2. Inclusion and Exclusion Criteria

All potential participants were assessed for eligibility.

Inclusion criteria included [i] being aged between 18 to 85 years; [ii] being able to understand the instructions associated with the study and to answer the questionnaires independently; [iii] being able to provide written consent, and being willing to comply with study requirements, namely completing questionnaires and not taking any other probiotic supplement for the 3-month duration of the study; and [iv] being on chemotherapy for gastrointestinal cancer after having been operated on.

Exclusion criteria included [i] being diagnosed for any psychiatric or neurological disorder or being on any antidepressant treatment, or having a history of a suicide attempt; [ii] already taking any type of probiotics, apart from yoghurt; [iii] study protocol violation, namely receiving less than 90% of the treatment, verified by the number of psychobiotics or placebo sachets returned; and [iv] already participating in another clinical trial.

### 2.3. Study Design and Participants

Patients were randomly assigned in a 1:1 ratio to receive either the psychobiotic regime or a placebo according to a computer-based table generated online [randomizer.org] under the supervision of the oncologist [E.C.], who was not further involved in the study process and data assessment. Randomization remained absolutely blinded to all other study coordinators. All participants received 4 packages—for the 4-week treatment—each containing 14 sachets of psychobiotics or placebo for the 7 days, and were instructed to take 1 sachet twice a day, diluted in a glass of tap water, two hours after eating. Sachets were identical in packaging, appearance, consistency, and solubility in drinking water, as well as in taste and smell—thanks to the kind preparation and donation of Uni-Pharma SA, Athens, Greece—thus, distinguishing between the placebo and the psychobiotics was impossible for both patient and physician.

A patient who forgot to take a dose could take it later the same day, or otherwise return the sachet at the follow-up appointment after treatment termination. A telephone-call the day before this appointment was made as a reminder. This was completed in order to exclude any patient who had not taken at least 90% of the doses. Additionally, in the middle of the treatment month, all patients were contacted either by phone or seen personally at their next chemotherapy appointment for a rough out-of-protocol communication. This contact, by the same physician who conducted the initial interview, aimed to confirm that the patients were taking the sachets and not experiencing any difficulties.

Finally, patients who only presented for the second psychometric assessment and who took more than the 90% of the doses of treatment, that is, at least 50 doses, were further evaluated.

### 2.4. Treatment Regimes

The psychobiotic formula consisted of 4 bacteria: *Bifidobacterium animalis* subsp. *lactis* LMG P-21384 [BS01] [2.50 × 10^10^ cfu/dose], *Bifidobacterium breve* DSM 16604 [BR03] [1.00 × 10^10^ cfu/dose], *Bifidobacterium longum* DSM 16603 [BL04] [8.00 × 10^9^ cfu/dose], and *Lacticaseibacillus rhamnosus* ATCC 53103 [GG] [4.50 × 10^10^ cfu/dose] to ensure a minimum microbial viable count of 1 × 10^9^ cfu/dose at the end of its shelf-life. The placebo contained Sorbitol, Steviol glycosides, Polyethylene Glycol 6000, and lemon–grapefruit flavors.

### 2.5. Participants’ Psychometric Assessment

Patients eligible to participate were contacted by a physician while waiting in the daycare center for their chemotherapy. This physician handed out paper and pencil versions of the assessment questionnaires, but was blinded to any individual’s group allocation. If necessary, patients could be helped by the physician, who could read out the questions and/or write down the dictated answers. Additionally, he/she collected patients’ demographics [age, gender, marital status, education level, yearly income, type of health insurance] and anthropometrics [body weight, height, and weight loss], as well as a short medical history relating to habits [smoking, alcohol, and sedative use] and a short history relating to the present disease: tumor location and invasiveness and co-morbidity indices (Charlson Comorbidity Index) as well as common side-effects of chemotherapy such as hair loss, diarrhea, vomiting, and chemotherapy temporary discontinuation due to hematologic profile disturbances.

Participants completed the questionnaires under the supervision of the same physician (to encourage a sense of familiarity) at all three defined time-points: at the first contact, upon joining the trial [T1]; 30 days thereafter, just after treatment termination [T2]; and two months thereafter, a total of 3 months from the first contact [T3].

### 2.6. Psychometric Tools Used

All the participants were evaluated at these three time-points by means of standardized psychometric questionnaires, which had been officially translated and validated in the Greek language by others in the past [48,49,50]. For the evaluation of the depression status, Beck’s Depression Inventory, 2nd Edition [BDI-II] [51], and the Hamilton Depression Rating 17-item Scale [HDRS] [52] were used. BDI-II is a self-report instrument first introduced by Beck et al. in 1961 and updated by him in 1996, the design of which was based on observations of attitudes and symptoms experienced mainly by patients with depression and, less frequently, by non-depressed individuals. The HDRS is a multi-dimensional tool for detecting a broad spectrum of clinical features associated with depression; it is considered a gold standard in depression studies and one of the most widely used observer-rated measures for the clinical evaluation of depression in clinical trials, with this version including 17 items serving as the standard. Anxiety was assessed by means of the General Anxiety Disorder-7 [GAD-7], which is a reliable and valid self-administered questionnaire developed by Spitzer et al. [53], evaluating generalized anxiety disorder symptomatology. Finally, the Perceived Stress Scale-14 Item [PSS-14] was used to evaluate patient’s stress status. The PSS-14 was introduced to assess stress from a psychological perspective, aiming to evaluate the degree to which individuals perceive situations in their life as excessively stressful relative to their ability to cope. It was first proposed by Cohen et al. in 1983 [54], and since then it has become the world’s most commonly used psychological instrument to measure perceived stress.

### 2.7. Sample Size Calculation and Power Analysis

A sample size calculation using the statistical program G*Power [Version 3] was performed based on bibliographic data on the prevalence of depression in cancer patients assessed with standard psychometric tools. Assuming that the overall prevalence of depression in patients with gastrointestinal cancer is up to 30% [55], independently of its severity, and by using a cut-off score of ≤7 [56] on the HDRS for discrimination between depressed and non-depressed participants, we hypothesized that the tested treatment should reduce the prevalence of depression in the participants by 50% based on the available literature [57] [i.e., the rate of depression in the psychobiotic group should decrease from 30% to 15% after treatment]. Based on these assumptions, we calculated that, for designing a superior trial with 80% power and a type I error rate of α = 0.05, a sample size of 120 patients per study-arm would be required [58]. Given the close monitoring of participants in this trial, we did not expect a dropout rate higher than 10% per arm; thus, we aimed for a total enrollment of 264 patients [132 patients in each arm].

An Intent-to-Treat (ITT) analysis approach was followed. The ITT method ensures that all patients who were initially randomized into the study, regardless of whether they completed the treatment or not, were included in the final analysis. One key advantage of ITT is that it preserves the benefits of randomization, helping to prevent bias that could arise from differential drop-outs between groups. This approach also enhances the generalizability of the results, as it reflects the real-world scenario where not all patients adhere to the treatment protocol. Additionally, ITT is considered a conservative analysis, as it tends to underestimate the true effects of the clinical intervention by including participants who may not have fully complied with the study protocol. As a result, it helps to avoid the potential overestimation of treatment effects that might occur if only those who completed the study were analyzed. By using ITT, we aimed to provide a more accurate and reliable estimate of the treatment’s effectiveness in a broader real-world context.

### 2.8. Data Collection and Statistical Analysis

All data collected were initially stored safely in a Microsoft Excel [Microsoft Office Professional Plus 2019] spreadsheet. The two investigators [G.T. and D.C.] responsible for entering data were both blinded regarding the patients’ randomization. Then, an .sav file was created for the purpose of editing by the statistician using the statistical software. Statistical analysis was conducted using the Statistical Package for Social Science (SPSS® 25.0, IBM Corporation, Armonk, NY, USA for Windows®/Apple Mac®), with the level of statistical significance set at 0.05.

The normality of the data distribution was assessed by performing the Kolmogorov–Smirnov test, given the fact that each group had more than 50 participants. For continuous variables, the results were reported as means ± standard deviation (SD) or as median and its accompanied interquartile range (IQR) when normality was either assumed or not, respectively. Categorical variables were presented as percentages. Student’s *t*-test or Wilcoxon test was performed in order for the means or medians, respectively, to be compared between the two independent groups at each evaluation. Moreover, repeated measures ANOVA or the Friedman test were used in order for the means or medians, respectively, to be compared between the three consecutive assessments within the same group. Regarding categorical data, statistically significant differences were detected by means of the Chi-square test. The McNemar test was used to determine whether there were differences in the dichotomous dependent variables between the two related groups. Moreover, the Risk Ratios (RRs) were calculated.

## 3. Results

### 3.1. Participant Characteristics

**Figure 1:**
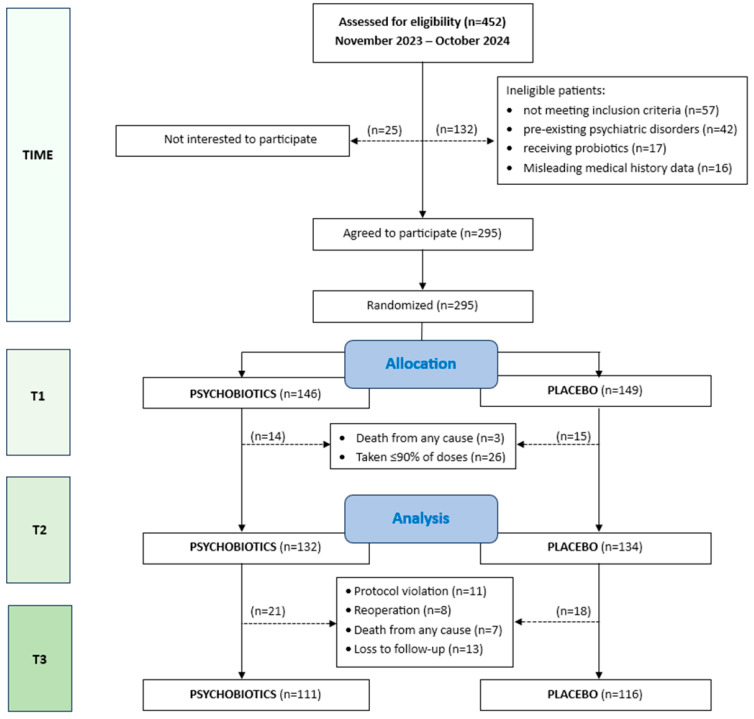
CONSORT flow diagram.

Four hundred and fifty-two patients on chemotherapy were initially screened for eligibility to enter the *ProDeCa* study. Full details of participants are reported in the CONSORT Flow Diagram [Figure 1]. Of the 452 candidates, 132 were ineligible, and 25 were not interested; the remaining 295 were initially enrolled and were randomized into the psychobiotics or placebo groups. Within the month of treatment there were three deaths, while another 26 participants returned more than six sachets/doses and were excluded from further assessment. Thus, a total of 266 participants [178 males, 88 females] remained for further analysis. According to protocol, participants should have completed at least the first two sets of questionnaires, that is, before [T1] and after treatment [T2], to finally be included.

Participant characteristics relating to demographics and anthropometrics, including habits, are summarized in Table 1A. It is of interest to note that more than 60% of the participants were married, had an under-diploma educational level, and a mid-level income of 10,000 to 30,000 euros. Table 1B summarizes information relating to medical history, the tumor location and invasiveness, the co-morbidity indices, and the side-effects of chemotherapy.

Overall, 21 [rate 15.9%] and 18 [rate 13.4%] patients in the psychobiotic and placebo groups, respectively, were lost at the 3-month follow up [T3]; these losses are analyzed to 4 and 3 deaths, 3 and 5 reoperations, 4 and 3 terminations of chemotherapy sessions, 2 and 4 refusals to complete the third course of questionnaires, and, finally, 8 and 3 continuations of the psychobiotic treatment with the commercially available product who were, thus, considered to be violating the protocol. We need to explain that this small number of patients [n = 11, still blinded], being very satisfied with their psychological improvement, asked for the name of the commercially available product in order to continue taking it. For ethical reasons, we did not feel able to refuse, although it would mean losing them to our study. Thus, the psychobiotics group at T3 had 111 patients instead of 132 and the placebo group had 116 instead of 134. When presenting the number of patients at T3, we insert a second number in parenthesis: this number represents the missing data extrapolated to the total number of patients.

### 3.2. Psychobiotics Downregulate Depression Status

From the total of 266 patients enrolled in the study, both BDI-II and HDRS psychometric tests revealed a total of 48 and 51 subjects out of the 266 studied [rates 36.4% and 38.1%, respectively] to be suffering from depression at different degrees of severity, with the male-to-female ratio being 2.8:1. Table S1 summarizes the depressed versus non-depressed patient characteristics in the psychobiotic and placebo groups; there is no significant difference between the two groups, so no parameter could be considered as a predisposing factor.

Since the BDI-II and HDRS psychometric tests have almost the same results, we decided from this point onwards to use only the results derived from the HDRS test. Thus, 99 depressed patients were allocated to the psychobiotic group [n = 48, in a ratio of 1:1.8 against the non-depressed] and to the placebo group [n = 51, in a ratio of 1:1.6 against the non-depressed].

Psychobiotics were found to significantly ameliorate the depression status of patients versus placebo treatment. Handling the result as a dichotomous variable [depressed and non-depressed, independently of the severity], there were 48 depressed individuals in the psychobiotics group at baseline [T1]; their number reduced progressively to 22 at the end of the treatment month [T2], and to 16 at the third-month follow-up [T3]. In other words, psychobiotic treatment switched 26 out of the 48 depressed patients into the non-depress status at T2, and then an additional 6 patients at T3, thus changing the ratio of non-depressed to depressed from 48 to 16 [relative improvement of 60.4%]. The RR at T2 was 0.18 (95% CI: 0.10–0.31), meaning that the risk of remaining depressed after psychobiotic treatment was 18% of the risk in the placebo group. At T3, the RR was 0.10 (95% CI: 0.05- 0.19). The opposite was the case in the placebo group, where the initial 51 depressed individuals increased to 71 [T2] and then to 73 [T3] [Table 2].

**Figure 2:**
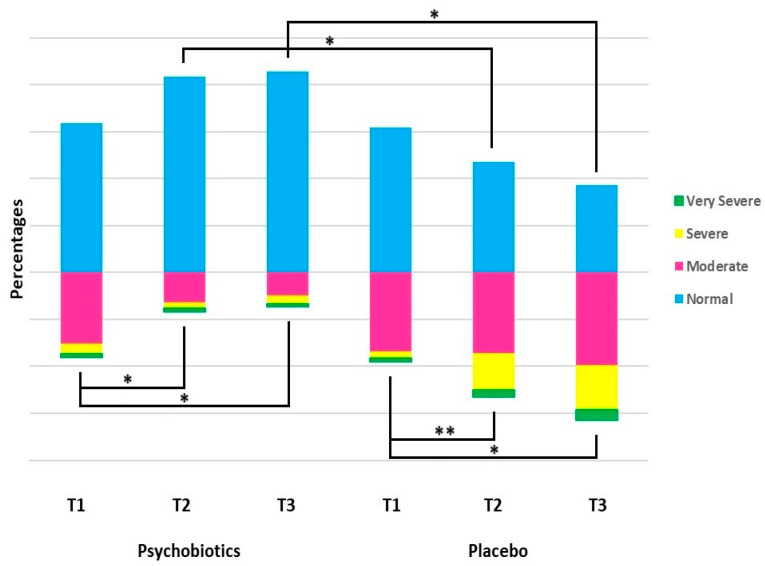
Distribution of participants according to depression status [HDRS scores: 0–7: Normal; 8–18: Moderate; 19–22: Severe; 23–50: Very Severe] in each group and at each time-point [T1: before treatment; T2: at treatment termination, one month; T3: at three months]. Data presented as percentages. Comparisons within the same group were made by means of McNemar test and between groups at each time-point by Chi-square test; * *p* < 0.001; ** *p* < 0.010.

The discrete fluctuations in depressed patient numbers according to the severity of depression over the time-period and according to treatment or otherwise [placebo], expressed in percentages, are shown in Figure 2.

### 3.3. Psychobiotics Improve Sleep Quality

By separately analyzing the questions relating to sleep quality and disorders of the BDI-II [question 16] and HDRS [questions Q4, Q5, Q6] tests, we studied the cases of insomnia and sleep quality in a dichotomous manner. To the question ‘I can sleep as well as usual’, the answers Yes/No were equal 1:1 at T1, while at T3 it improved (the Yeses) to 1.8:1 in the psychobiotics group, but fell to 0.9:1 in the placebo group. Regarding insomnia, the answers No/Yes for the ‘Insomnia early in the night’, meaning difficulty falling asleep, were 1:0.8 at T1 and improved to 3.6:1 in the psychobiotics group at T3. For the placebo, the ratio was 1:1 at T1 and deteriorated to 0.3:1 at T3. For the answer No/Yes for the ‘Insomnia middle of the night’, meaning waking during the night or being restless during the night, the ratio 0.7:1 in the psychobiotics group at T1 improved to 2:1 at T3. Correspondingly, for the placebo, it was 0.8:1 and 0.7:1, respectively.

**Figure 3:**
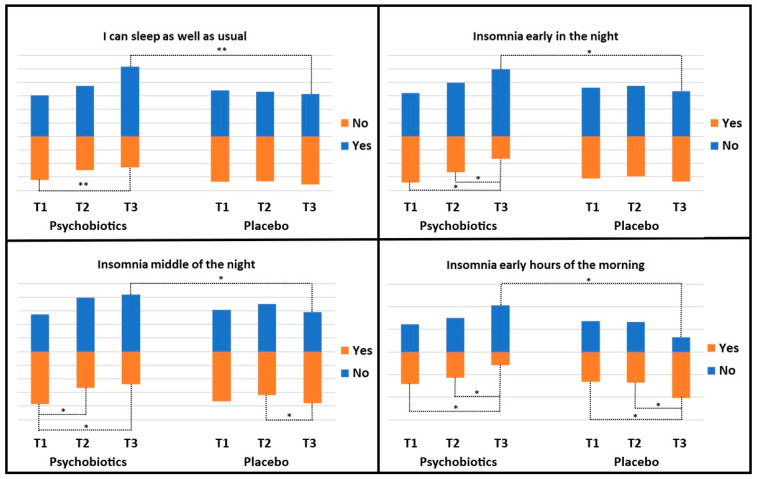
Distribution of participants according to dichotomous classification of sleeping disorders [yes or no] in each group and at each time-point: T1: before treatment; T2: at treatment termination, one month; T3: at three months. Data presented as percentages. Comparisons within the same group were made by means of McNemar test and between groups at each time-point by Chi-square test; * *p* < 0.001; ** *p* < 0.05.

Finally, for the answer No/Yes for the ‘Insomnia early hours of the morning’, meaning waking in the early hours in the morning and being unable to fall asleep again, the ratio was 1:0.9 at T1 and improved to 2.9:1 and 1.2:1, which slightly deteriorated to 1:1 at T3 in the psychobiotics and placebo groups, respectively. A detailed illustration of these changes over time is also added in Figure 3.

### 3.4. Psychobiotics Improve Anxiety Status

The assessment of anxiety was made using the GAD-7 scale questionnaire. To discriminate between individuals with and without anxiety in a dichotomous manner, the validated cut-off value of ≥8 was used; half of the participants [n = 138, rate 51.9%] were experiencing anxiety. These were equally distributed between the psychobiotics group [n = 72, rate 54.5%] and the placebo group [n = 66, rate 49.2%]. Regarding age, gender, socioeconomic status, and education level, there was no clear difference between the anxiety and non-anxiety individuals [data not presented].

Psychobiotics were found to significantly improve anxiety: 54.5% of the treatment group [72 patients] at baseline was reduced to 36.4% [n = 48] after a month of taking psychobiotics and then further to 23.4% [n = 26 in a total of 111 patients presenting for follow-up] at the third month, that is, two months after treatment termination [relative improvement of 57.0%]. The RR at T2 and T3 was 0.30 (95% CI: 0.18–0.49) and 0.13 (95% CI: 0.07–0.24), respectively. However, the anxiety rates for the placebo group were found to progressively increase [relative deterioration of 39.7%]: 49.2% [n = 66 participants out of 132] at T1, 65.7% [n = 88 out of 132] at T2, and 69.8% [n = 81 out of the 116 who presented for follow-up at 3 months] at T3, respectively [Table 3].

**Figure 4:**
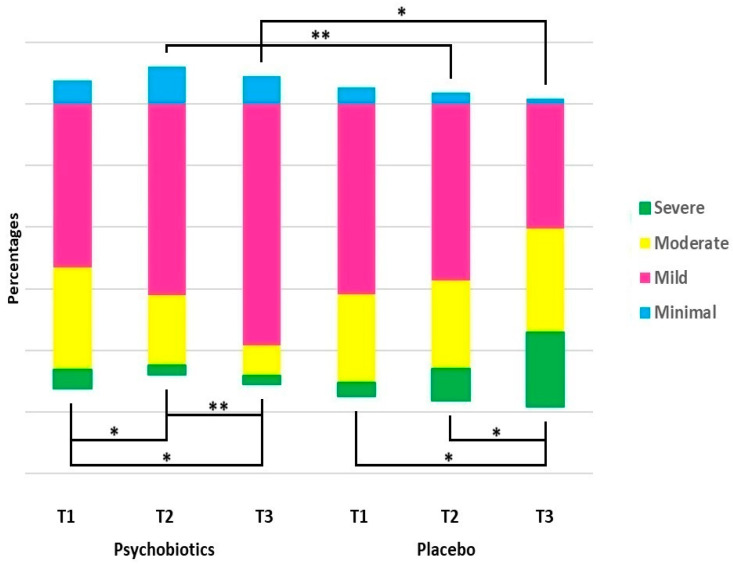
Distribution of participants according to severity of anxiety [GAD-7 scores: 0–4: Minimal Anxiety; 5–9: Mild Anxiety; 10–14: Moderate Anxiety; >15: Severe Anxiety] in each group and at each time-point: T1: before treatment; T2: at treatment termination, one month; T3: at three months. Data presented as percentages. Comparisons within the same group were made by means of McNemar test and between groups at each time-point by Chi-square test; * *p* < 0.001; ** *p* < 0.010.

The analysis of patients according to anxiety severity and consequent changes in relation to treatment or placebo over time are depicted in Figure 4.

### 3.5. Psychobiotics Improve Perceived Stress

**Figure 5:**
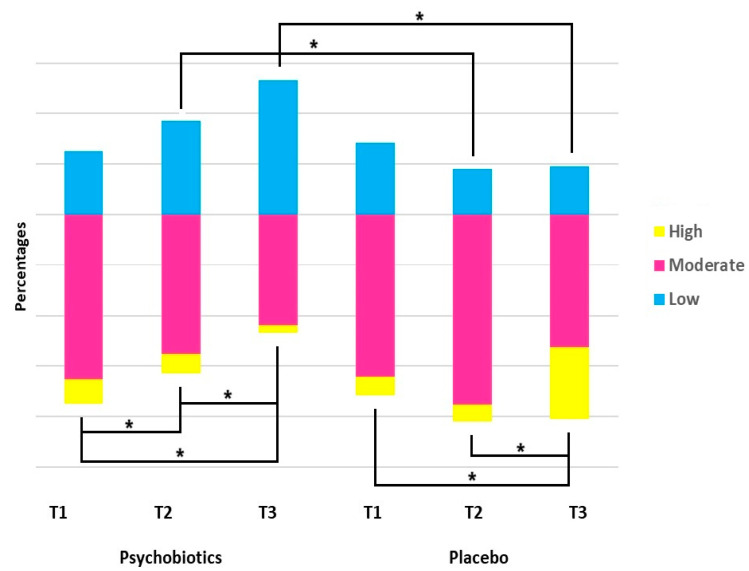
Distribution of participants according to stress status [PSS-14 scores: 0–18: Low Stress; 19–37: Moderate Stress; 38–56: High Stress] at each time-point and for each treatment group. T1: before treatment; T2: at treatment termination, one month; T3: at three months. Data are presented as percentages. Comparisons within the same group were made by means of McNemar test and between groups at each time-point by Chi-square test; * *p* < 0.001.

For the assessment of stress, the PSS-14 was used, with scores ranging from 0 to 56. Low stress is characterized by scores between 0 and 18, which applied to 71 patients out of the 266 [rate 26.7%]. These ‘low-stress’ individuals were almost equally [*p* = 0.536] divided between the psychobiotics [n = 33] and placebo [n = 38] groups. After taking psychobiotics, the number of ‘low-stress’ participants increased to 49 [at T2] and 59 [at T3], while the placebo group had a decrease in the initial number from 38 to 24 [at T2] and then to 22 [at T3]. In other words, psychobiotics almost doubled the percentage of low-stress individuals from 33.3% to 53.1% [at T3] [*p* < 0.001], while for those taking the placebo, it decreased from 28.3% to 18.9% [at T3] [*p* = 0.043]. The results of the analysis of participants according to the PSS-14 score into three stress status levels [low, moderate, and high] at each time-point and for each treatment group are illustrated in Figure 5.

When the cut-off value of ≥28 [59] was used to simply dichotomize participants into No/Yes stress, the number of stressed patients taking psychobiotics was 42 [at T1], 27 [at T2], and 14 [at T3], presenting a significant decline [*p* = 0.001] in relation to the placebo-treated patients [relative improvement of 60.4%]. The RR at T2 and T3 was 0.51 (95% CI: 0.29–0.89) and 0.06 (95% CI: 0.03–0.12), respectively. For placebo-treated patients, stress progressively increased from 38 at T1 to 45 at T2 and then to 81 at T3 [*p* < 0.001] [relative deterioration of 142.5%], Table 4.

## 4. Discussion

In the present study, we clearly accept the ability of this psychobiotic formula to downregulate the stress, anxiety, and depression status of patients undergoing complementary chemotherapy after a radical dissection for gastrointestinal cancer. Moreover, our encouraging results—in terms of emotion and mood status enhancement—are further reinforced by the negative findings in the placebo group: this group, consisting of patients who received placebo treatment, was found, as expected, to have a negative improvement on the psychometric parameters studied. This group had no psychological support apart from the “placebo effect”, if such an effect really exists. However, according to the position statement on antidepressants issued by the Royal College of Psychiatrists, an improvement in depression symptoms is achieved at a rate of 50% to 65% for those patients receiving an antidepressant, and 25% to 30% for those taking a placebo [60].

Psychobiotics are defined as the probiotics which, when taken in adequate quantities, exert a health benefit on patients suffering from psychiatric illness by means of their unique characteristics of producing and delivering neuroactive substances which can influence the complex bidirectional microbiota–gut–brain axis [61]. The recognition of the involvement of gut bacteria in mood control led to the idea of manipulating the gut microbiota to produce benefits for brain function. From this point of view, the term ‘psychobiotic’ expresses any exogenous intervention that leads to a bacterially mediated impact on the brain [62]. An early study, almost the first, reporting the distinct impact of probiotic administration at behavioral, neural, and microbiome levels at the same time in healthy volunteers was that of Bagga et al. [63]. The authors revealed—by means of functional MRI—that a 4-week probiotic formula taken by healthy adults was directly related to changes in brain activation patterns, showing a significant difference in brain activity in the cingulum, precuneus, inferior parietal lobule, thalamus, and para-hippocampal gyrus. Additionally, the subtle modifications in the gut microbiome profile as well as behavioral improvements in memory and decision-making processes, especially in the context of emotions, triggered future investigations into the role of probiotics in the context of major depression and stress disorders [64].

Today, it is well known that gut bacteria communicate with the central nervous system by triggering the vagus nerve by producing antigens recruiting inflammatory cells, and by entero-endocrine signaling from the intestinal epithelial cells [65,66,67], to regulate neuroinflammation, neurogenesis, neurotransmission, and neuroendocrine signaling, by means of producing or being involved in the induction of neurotrophic factors and neurotransmitters, including γ-aminobutyric acid, dopamine, serotonin, glutamate, and the brain-derived neurotrophic factor (BDNF). All of these play a crucial role in governing the neural excitatory–inhibitory balance, mood, cognitive functions, learning and memory processes, and sleep disorders, among others [68,69]. On this basis, it has been suggested that the modification of gut microbiota may have translational applications in the modulation or even treatment of neuropsychiatric illnesses [39,70,71,72].

Experimental and clinical studies of bacteria with psychotropic effects—namely psychobiotics—have tended to concentrate on those belonging to the Bifidobacterium and Lactobacillus families. This does not, however, exclude other species, less studied as yet, such as *Escherichia coli Nissle* 1917, *Akkermansia muciniphila*, *Faecalibacterium prausnitzii*, and *Clostridium butyricum* [73]. In the present study, a psychobiotic formula containing *Bifidobacterium animalis* subsp. *lactis* LMG P-21384 [BS01], *Bifidobacterium breve* DSM 16604 [BR03], *Bifidobacterium longum* DSM 16603 [BL04], and *Lacticaseibacillus rhamnosus* ATCC 53103 [GG] at a concentration of 1 × 10^9^ cfu/dose was used. These four probiotics are part of a commercially available formula also containing magnesium and saffron [LactoLevure^®^ ProbioMood, Uni-Pharma, SA, Athens, Greece], well known for their antidepressant properties [74] and marketed for ‘people with an anxious lifestyle, who often suffer from symptoms such as low mood or fatigue as a result of stress’. Since the research in psychobiotics covers only a short period of 10 years, it is evident that studies should investigate new species or strains, rather than repeat the use of the same ones, which means that extended research on particular bacteria is rather limited.

Regarding the bacteria included in the formula we used, we know the following: in a previous study, the same four-probiotic formula was used in concentrations which differed to ours, and it was found to increase *Bifidobacteria* counts and, thus, to improve sleep quality, general fatigue, anxiety, and cognitive symptoms [75]. *B. animalis* subsp. *lactis* LMG P-21384 [or *B. lactis* BS01] is known for its action in preventing and/or treating inflammatory processes—major surgery for cancer being such a serious process—by means of the highly effectively immune stimulation of the polarization of monocyte/macrophage subpopulations. Additionally, it was found to significantly mitigate oxidative stress [76], while *B. lactis* BS01 (2 × 10^9^ cfu) and *L. acidophilus* LA02 were tested, prophylactically, in 50 healthy females to investigate whether it would exert benefits in cognitive functioning [77].

*B. breve* DSM 16604 [BR03] was found—by means of HPLC coupled with mass spectrometry—to have the ability to secrete high concentrations of acetic acid in the culture supernatant [78]. Although its exact role has not yet been fully demonstrated, it is known that it is widely effective in ameliorating obesity-associated insulin sensitivity by directly or indirectly modulating the local concentration of short-chain fatty acids [SCFAs] [79]. In addition to all the other well-known beneficial effects, like the maintenance of gut barrier integrity, the SCFAs have an impact on the production of neurotransmitters in the brain. Acetate has been documented to increase the hypothalamic levels of g-aminobutyric acid [GABA] and lactate in vivo, while in vitro studies have shown that it modulates serotonin levels by regulating the expression of tph1, which encodes tryptophan 5-hydroxylase 1, the enzyme necessary for serotonin production [80,81]. Another study showed the effects of *B. breve* BR03 along with *S. thermophilus* FP4 on stressed athletes, undergoing periods of intense training; the catabolism of tryptophan via kynurenine seems to play an important role in alleviating the transient suppression of the immune function due to prolonged intense exercise [82]. Thus, a 3-week treatment prior to muscle-damaging exercise may assist in performance recovery, reducing the inflammation subsequent to strenuous exercise [83]. Benefits were also reported for the improvement in the aerobic capacity of male soccer players [84].

*B. longum* DSM 16603 [BL04], along with *L. fermentum* LF16, *L. rhamnosus* LR06, and *L. plantarum* LP01, exerts a significant improvement in mood status, with a reduction in depression, anger, and fatigue, as well as an improvement in sleep quality; the effect was maintained 3 weeks after washing-out treatment [85]. Similarly, BL04 [along with *L. rhamnosus* HN001 and *B. infantis* Bi-26], given to children with an autism spectrum disorder [86], was reported to improve atypical emotional behavior and neurological dysfunction [87].

*L. rhamnosus* ATCC 53103 [LGG] is one of the most widely used probiotic strains in the world, and the health-promoting effects of this bacterium have been well documented in clinical and animal studies [88,89,90]. The oral administration of LGG alters autonomic neurotransmission, including adrenal sympathetic nerve activity, gastric vagal nerve activity, and cutaneous arterial sympathetic activity, in a dose- and strain-dependent manner [91]. It was also found that LGG positively influences hippocampal neuronal viability, along with synaptic and functional development, as well as the regulation of neuronal BDNF protein, when added to a hippocampal neuron culture [92]. Cryan’s group, who are working on the established rat model of maternal separation for early-life stress induction, which is known to result in depression-like behaviors in adulthood [93], initially found a differentiation of the gut microbiota versus control rats, suggesting a mechanistic role for the microbiota–gut–brain axis. They then fed the rats with LGG, with or without prebiotics, and found that those who received LGG plus prebiotics exhibited a reduction in anxiety-like behavior and hippocampal-dependent learning due to alterations to the hippocampal mRNA expression of genes related to stress circuitry, anxiety, and learning [94]. In a rat model of Celiac disease, the brain-derived neurotrophic factor [BDNF]—known to act as the bridge between immune activation and the nervous system’s adaptive response—was found decreased, which was associated with psychiatric comorbidities; ten days of treatment of rats with LGG [10^9^ CFU/day] was found to positively affect BDNF levels [91]. Furthermore, LGG was found to be the major contributor to the induction of neurotrophic factors and the downregulation of monoamine oxidase B in relation to *B. animalis lactis* (BB-12) and *L. acidophilus* (LA-5). Regarding the mechanism of action, it seems clear that these specific probiotics can rescue the dopaminergic neurons by means of increasing butyrate production and, thus, increasing neurotrophic factors [95]. Finally, it was found that LGG secretes high concentrations of acetic acid [78], which has already been analyzed in relation to *B. breve* DSM 16604 [BR03].

In the present study, we used two different tools to identify and evaluate depressed individuals: the Beck’s Depression Inventory [BDI] [51] and the Hamilton Depression Rating Scale [HDRS] [52]. We found a total of 48 and 51 subjects out of the 266 studied [rates 36.4% and 38.1%, respectively] suffering from depression of differing degrees of severity. This rate is, to some degree, consistent with the current bibliography; depression is estimated to affect approximately 25% of cancer patients, five times more than the general population [96]. Mitchell et al. [97] referred to percentages ranging from 4% to 60% in oncological, hematological, and palliative care patients, while Massie [98] to a rate of 38% for major depression and of 58% for depression spectrum syndromes. In 200 Chinese patients with gastric cancer, the prevalence was 33.5%, analyzed into 9.0%, 41.8%, and 49.3% in respect to severe, moderate, and mild depression [10]. In advanced colon cancer patients in Brazil, the prevalence of depression was 44.3% and of anxiety 25.7%, while 52.9% of patients had both [99]. In China, on the other hand, prevalence was only 11% for depression and 29% for anxiety [100]. Reported variances are due to differences in stage and tumor site, but should always take into consideration the emotions of individuals, which may relate to the geographical background, and the higher rates generally conform towards the end of a patient’s life [101]. Our patients, although extroverted characters as typical of Mediterranean origin, were found to have a rather high prevalence of depression. Another point, however, is that our study deals with cancer patients being diagnosed with cancer, which was the first hit; being operated on for the cancer, which usually left externally visible changes on their bodies, i.e., long surgical incision scars and external stomas, which was the second hit; and then undertaking the long process of a chemotherapy course, which, besides the tissue destruction itself, can additionally have severe adverse effects [102], which was the third hit.

Despite all these negative impacts, it is of interest to underline the significant amelioration in depression status in the group who received psychobiotics versus the controls: at baseline [T1], there were 48 depressed individuals in the psychobiotics group and 51 in the control group. After the month on psychobiotics, the number of depressed patients was highly significantly decreased [from 48 to 22] and patients moved to a less severe status, while no suicide cases were identified in either group. Kamimura et al. [101], in 109 cancer patients assessed by psychometric questionnaires three times before finishing chemotherapy, found that their scores were the highest on commencing chemotherapy and declined thereafter. However, in 100 breast cancer females, the pre-chemotherapy depression severity was at a minimum level, but after the onset of therapy, it significantly increased [18]. This is consistent with our findings, since the placebo-treated group presented with a progressive increase in the number of depressed individuals, mainly due to the significant side-effects, i.e., diarrhea, hair loss, etc., negatively affecting quality of life and/or the prolongation [34,103] or the non-response to treatment [104]. Furthermore, it is known that patients receiving chemotherapy sessions with a short-term free interval and for a long period are considered to be more susceptible to systemic inflammations, perhaps due to the incomplete recovery of microbiota between treatments, and which could also contribute to increased depression symptoms [34].

Today, it is well known that psychobiotic supplementation significantly alleviates mood disorders due to its ability to produce tryptophan, assisting in serotonin synthesis by the host [105,106,107,108,109]. These positive effects of psychobiotic bacteria, mainly bifidobacteria, have been observed not only in depressed individuals, but also in healthy individuals: 4 weeks of *B. longum* W23 and W52 was found to reduce negative thoughts related to sad mood [110]. Similarly, in a 12-week clinical study of 32 volunteers self-defined as “non-stressed”, the administration of a symbiotic formula containing *B. animalis* subsp. *lactis* and *L. paracasei* HII01, as well as galacto- and fructo-oligosaccharides, was found to significantly reduce tryptophan levels, while increasing, among other things, the amount of acetate and propionate [111]. Zhang et al. [112], in a recent meta-analysis of 13 studies, confirmed that the manipulation of the gut microbiota by the appropriate bacteria, namely psychobiotics, could become the new approach to treating patients with mild to moderate depression. However, it is of interest that studies in which the percentage of females was under 70% reported a larger reduction in depression scores, and in our study, the male-to-female ratio was 3:1 [73 males: 26 females] against 1.7:1 in the non-depressed. In another meta-analysis of 10 RCTs dealing with the effects of psychobiotics versus placebo in patients suffering Major Depressive Disorder, the authors conclude that psychobiotics have great potential in treating depression, with the results positively correlating with treatment duration, but no ideal specific strain, dosage, or treatment duration currently stand out [41].

The pathophysiology of depression in relation to gut microbiota was recently reconfirmed. *Bifidobacterium* and *Lactobacillus* counts estimated in fecal samples revealed significantly lower *Bifidobacterium* and a trend of lower *Lactobacillus* counts in patients with depression versus controls [113]. A meta-analysis involving 1519 individuals experiencing depression, anxiety, and other psychiatric disorders revealed consistent differences in gut microbiota diversity. Those with depression had reduced levels of the anti-inflammatory bacteria *Faecalibacterium* and *Coprococcus*, and increased levels of pro-inflammatory molecules in relation to healthy controls, thus suggesting that depression is characterized by a depletion of bacteria producing anti-inflammatory compounds like SCFAs, and, simultaneously, by an enrichment of pro-inflammatory microbial genera [114,115], implicated, among others things, in the modulation of tryptophan metabolism [116].

Biologically, inflammation has been extensively discussed for its association with depression in patients with medical illness, and is especially relevant to cancer, since many aspects of cancer induce inflammation. Apart from the cancer itself, all related manipulations—surgery, chemotherapy, radiotherapy, immunotherapy—which induce significant tissue damage and destruction also result in excess inflammation [101]. Inflammatory cytokines and other molecules can drive immunological alterations in the brain [11], and can affect the activity of the HPA axis, as well as every aspect of the metabolism of monoamines, including serotonin, norepinephrine, and dopamine, all crucial in the regulation of mood [33,117], and thus leading to anxiety, depression, and other psychiatric disorders [118].

Among the disorders related to depression is insomnia, although it is now considered as separate entity. Indicative of its seriousness is that the HDRS questionnaire dedicates 3 out of the 17 items to insomnia [and two to anxiety], while all other parameters of depression have 1 question each. Half of our participants, based on the question of the BDI-II test ‘I can sleep as well as usual’, were found to suffer from insomnia. However, when their ‘yes’ was analyzed based on the questions of the HDRS, that is, insomnia early or in the middle of the night or the next morning, we recognized that a small number of patients experienced at least one type. By taking psychobiotics, the ratio yes/no for the question ‘I can sleep as well as usual’ improved from 1:1 to 1.8:1. This became much better for the ’early in the night insomnia’: no/yes from 1:0.8 at T1, which improved to 3.6:1 at T3; for the ‘middle of the night’, from 0.7:1 to 2:1; and for the ‘early hours in the morning’, from 1:0.9 to 2.9:1.

However, placebo-treated participants exhibited a deterioration, probably related to the progress of the cancer and its complications, as well as the increase in the severity of depression, anxiety, and perceived stress. Sixty anxious students took *L. plantarum* JYLP-326 or a placebo twice a day for three weeks. Insomnia was significantly decreased in the probiotic group, while it was marginally increased in the placebo group at the end of treatment, with the severity of insomnia being bidirectionally associated with anxiety and depression [3,119]. In the same manner, the *B. breve* CCFM1025 was found to improve sleep quality by enhancing stress-induced insomnia patients’ subjective sleep quality and reducing sleep disturbance [particularly early waking at night], along with a more pronounced reduction in stress markers in relation to controls [120]. According to Haarhuis et al. [121], neural, endocrine, and immune pathways, as well as clock gene regulation, are involved in the sleep-promoting and wakefulness-promoting effects of psychobiotics. Additionally, besides their direct effects, beneficial microbiota can also influence these processes via the production of SCFAs. Two out of the four psychobiotics used in our study are documented to produce SCFAs, *B. breve* BR03 and *L. rhamnosus* [LGG], while *B. longum* BL04 have previously been confirmed to have positive effects on sleep disorders [85].

Regarding anxiety, in the present study, we identified more than half of our patients as suffering from anxiety according to the diagnosis made by the GAD-7 scale questionnaire [53,122]. In more detail, and by using ≥8 as the cut-off point, there were 72 out of 132 patients [rate 54.5%] in the treatment group and 66 out of 134 [rate 49.2%] in the placebo group. Anxiety in general is considered as a “normal” potentially adaptive psychological reaction in cases deemed to be life-threatening; but, it becomes a clinical issue when it is dominant and overarching and severity and/or duration surpass the normal expectations [2,24]. Then, it may become a generalized anxiety disorder, manifested by psychological arousal symptoms, such as sleeplessness, restlessness, and muscle tension, along with excessive worry [123]. Pandey et al. [14] splits anxiety into four types: situational anxiety, disease-related anxiety, treatment-related anxiety, and as an exacerbation of pre-treatment anxiety disorder.

Several studies have reported a prevalence of between 12% and 25% or even over 40% in oncological patients undergoing surgery [20,22,23,24]; anxiety is likely to be higher because of additional fears of the unknown, of response to tumor surgery, pain, loss of control over the situation, dysfunction, alienation, loss of autonomy, increased rates of complications and, thus, hospital readmissions, and perhaps of the perspective of life or even death [3,4,124]. Harms J et al. [3] reported a rate as high as 56% of mild or moderate anxiety in such a group of 101 patients, although the authors do comment that anxiety levels do not behave statically but dynamically during the perioperative course and are therefore less predictable. This ratio is closer to that of our study cohort; it must not be forgotten, however, that our subjects were chemotherapy patients who had already been subjected to gastrointestinal surgery a short time before. Although chemotherapy alone is considered to cause less anxiety than oncological surgery [3,125], the combination of the processes in sequence, and taking into consideration that chemotherapy is an intense and cyclic treatment associated with a number of side-effects [14], having a dramatic impact on patient mood should be considered probable.

As with depression, anxiety is generally more common in females, in patients older than 35 years, and in those of low socioeconomic status and of low education level [21,126]. However, a recent meta-analysis of 6317 cancer patients reported the opposite: males experienced significantly higher levels of anxiety than females, while the opposite was the case for depression [127]. In our patients, the male-to-female ratio was found to be almost equal. The reason for the gender difference could be attributable to the males having different perceptions and coping strategies to females for dealing with the ‘accident’ of cancer and the subsequent surgery. The female anxiety response could be exacerbated by their social role in the family network or the partnership [3], although, ultimately, how any individual responds will relate to their individual inherent predisposition to handle the disease condition they face [20,128,129].

Psychobiotics were found to significantly improve anxiety: 54.5% of those anxious in the treatment group [72 patients] at baseline was reduced to 36.4% after a month of receiving psychobiotics, and then further to 23.4% at the third month, that is, two months after treatment discontinuation. The rates for the controls, however, were 49.2%, 65.7%, and 69.8%, respectively, clearly highlighting the benefits psychobiotics. One could negatively comment on the gradual increase in anxiety rates in the placebo group, since the literature indicates that patients were more anxious at baseline and gain greater psychological stability later [130]. However, this can be easily explained by the extension in the number of chemotherapy sessions, by possible complications, by increasing frailty and the loss of muscle mass, and generally of the poor quality of life.

Along with anxiety and depression, participants were found to live in chronic stress following cancer diagnosis, and subsequence treatments trigger many additional stressors, such as fear of death, interruption of life plans, changes in body image and self-esteem, and changes in social role and lifestyle [131]. Stress, even at a low level, induces excessive corticosterone and adrenocorticotropic release [132], which, although beneficial in the short term by helping the body to fight the stress trigger, when continuous, it can suppress the effects of the acute response and lead to low-grade inflammation [133]. Stress as a chronic condition is considered capable of changing neuronal morphology, suppressing neural proliferation, and reducing synaptic plasticity and neuron firing properties. Finally, it may lead to a reduction in the volume of the hippocampus with, as a consequence, the disturbance of memory, learning, and cognitive functions related to the hippocampus [134].

A considerable proportion of our patients reported perceived stress during chemotherapy, as assessed by the PSS-14 score, used to evaluate the degree to which situations in the participant’s life within the last month are appraised as stressful: 42 out of the 132 in the psychobiotic group and 38 out of 134 in the placebo group. At the end of the 3-month study, psychobiotics were found to have ameliorated stress, so that only 14 participants remained stressed. In the placebo-treated group, however, the 38 stressed participants increased to 81 at T3. In a clinical study [107], 45 healthy adults were allocated to a psychobiotic or control diet for 4 weeks. The psychobiotic diet resulted in a 32% reduction in perceived stress, the higher the adherence, the stronger the decrease, compared to only 17% with the control diet. In parallel, the psychobiotic diet induced significant changes in 40 specific fecal lipids as well as in urinary tryptophan metabolites. Restraint stress in adulthood in rats subjected to maternal separation in the first 10 days after birth resulted in exaggerated corticosterone plasma levels and increased stress-induced visceral sensitivity; LGG treatment improved beta diversity, while 41 genes in the spinal cord were downregulated after treatment, 12 of which were associated with stress, pain, or both [94].

This study has some limitations: first of all, we were not able to evaluate biological samples from the participants. It would have been very useful to have measured cortisol in saliva and tryptophan metabolites and/or microbiota in feces. Unfortunately, this was not possible due to the likelihood of refusal of a large percentage of participants. Second, we could have had double the number of participants; however, we thought it preferable to restrict the study to patients with gastrointestinal cancers in order to gain homogeneity, since a laparotomy and its possible complications is possibly the most stressful forerunner following chemotherapy. Third, treatment lasted only a month, although antidepressant formulas are classically given for 3 months to achieve some positive effects. We took this risk, although we had found only one study which mentioned the beneficial effects of psychobiotics lasting for 3 weeks after washing-out treatment [85]. Relative to this is the short—3 months only—follow-up. However, we are dealing with patients suffering from cancer and, thus, this would likely be impossible, taking into consideration that, within two months, from T2 measurement to T3, we lost 39 participants. Fourth, since depression is related—in many cases—to feeding refusal and, thus, to malnutrition and/or sarcopenia, one could comment that our participants should had been nutritionally assessed before recruitment. However, the main point of this study was to evaluate the beneficial effect of psychobiotics on depressed individuals, their depression status depending on their “disease” and other co-factors, including cancer-related malnutrition and frailty, and not to relate depression with any poor nutritional status. Fifth, one could comment that the dose of psychobiotics given is low [four bacteria at a concentration of 1 × 10^9^ cfu/dose, twice a day] based on the principle ‘as many strains/as high doses as possible’ [121]. Finally, another suggestion could be to split the participants into four subgroups and treat each one with one of the strains used to determine their effectiveness. This has a logical background, but ignores all the synergic effects of the four psychobiotics, two of which are known to release SCFAs too.

## 5. Conclusions

Our findings from this ProDeCa study strongly suggest that, in patients on a course of chemotherapy following gastrointestinal surgery for cancer, depression and anxiety, as well as perceived stress and sleep disorders, are a severe burden. More than half of the study population had at least one of these psychiatric disorders, diagnosed by the use of standard psychometric assessment tools, and which, in most of the cases, had remained otherwise undiagnosed. Psychobiotics taken twice a day for a month affected a highly significant improvement in all parameters, in parallel with a significant decline in the placebo group, as the 3-month study period progressed. Our results suggest the benefit of early assessment and psychobiotic support in oncological patients, even before surgery. However, further research is required both to elucidate in depth the specific mechanisms and to test the reproduction of results in different carcinoma groups or produce better results in relation to different concentrations of psychobiotics or to different strains. We are already working to this end.

## Figures and Tables

**Table 1:** (A). Participants’ baseline demographics, anthropometrics, and habits. (B). Patients’ baseline characteristics related to tumor location and invasiveness, co-morbidity indices, and the side-effects of chemotherapy.

(A)			
	Psychobiotics	Placebo	*p*-Value
**Number of patients**	132	134	
Age (years) *	69.5 (17.0)	65.0 (15.0)	0.061
Gender (Male/Female)	88/44	90/44	0.931
Weight (kg) **	74.8 (14.9)	74.9 (18.0)	0.754
Height (cm) **	170.7 (8.2)	170.4 (9.0)	0.856
BMI (kg/m^2^) **	25.7 (4.8)	25.9 (4.8)	0.816
Weight Loss (Yes/No)	104/28	96/38	0.177
**Marital Status**			0.265
Free	22	22	
Married	84	90	
Divorced/Widow/er	14/12	6/16	
**Education**			0.401
Basic	30	35	
Secondary	70	60	
University	32	39	
**Income**			0.263
EUR < 10 K	42	41	
EUR 10–30 K	84	80	
EUR > 30 K	6	13	
**Health Insurance**			0.297
Public/None	120/11	117/4	
Private	2	2	
Both	4	11	
**Habits**			
Smoking (Yes/No)	54/78	46/88	0.395
Alcohol (Yes/No)	16/116	26/108	0.103
Sedatives (Yes/No)	32/100	24/110	0.205
**(B)**			
	**Psychobiotics**	**Placebo**	***p*** **-Value**
**Number of patients**	132	134	
**Tumor Location**			0.232
Gastric	32	33	
Large Bowel	65	64	
Rectum	24	27	
Pancreas	11	10	
**Disease stage**			0.571
Stage I	16	15	
Stage II	58	54	
Stage III	46	51	
Stage IV	12	14	
Charlson Comorbidity Index *	5.0 (1.0)	5.0 (1.0)	0.212
**Side-effects of chemotherapy**			
Diarrhea (Y/N)	34/98	37/97	0.732
Vomiting (Y/N)	17/115	21/113	0.515
Chemo—temporary discont. (Y/N)	17/115	14/120	0.382
Hair Loss (Y/N)	13/119	12/122	0.803

(A): * Data presented as median and interquartile range [IQR], or ** as mean ± standard deviation. Age was assessed by means of Mann–Whitney U test, anthropometric data [weight, height, BMI] by Student’s *t*-test for intendent samples, and all other parameters by the Chi-square test. (B): * Data presented as median and interquartile range [IQR], Charlson Comorbidity Index was assessed by means of Mann–Whitney U test, and all other parameters by the Chi-square test.

**Table 2:** Psychobiotics improve depression status: the number of non-depressed participants (HDRS) progressively increased, whereas for those taking a placebo, it decreased.

	Psychobiotics			Placebo		
Patients	T1 *	T2 ^a^	Τ3 ^b^	T1 **^, ‡^	T2	Τ3
Non-Depressed	84	110	95 (113)	83	63	43 (50)
Depressed	48	22	16 (19)	51	71	73 (84)
*total number*	132	132	111 (132)	134	134	116 (134)

* *p* < 0.001 between T1 and T2, and T1 and T3 within the same group (Mc Nemar test); ** *p* = 0.002 between Τ1 and T2 within the same group (Mc Nemar test); ^‡^ *p* < 0.001 between T1 and T3 within the placebo group (Mc Nemar test); ^a^ and ^b^ *p* < 0.001 between psychobiotics and placebo at T2 and T3 (Chi-square test). Parentheses represent the missing data, extrapolated to the total number of patients.

**Table 3:** Psychobiotics improve anxiety status: the number of non-anxious participants (GAD-7 score) progressively increased in the psychobiotics group, whereas for those taking the placebo, it decreased.

	Psychobiotics			Placebo		
Patients	T1 *^, ‡,^ **	T2 **^, a^	Τ3 ^b^	T1 ^†^	T2	Τ3
Non-Anxious	60	84	85 (101)	68	46	35 (40)
Anxious	72	48	26 (31)	66	88	81 (94)
*total number*	132	132	111 (132)	134	134	116 (134)

Comparisons within the same group were made by means of McNemar test: * *p* < 0.001 between T1 and T3 within the same group; ^‡^ *p* = 0.009 between Τ1 and T2 within the same group; ** *p* = 0.002 between T1 and T3 within the same group; ^†^ *p* < 0.001 between T1 and T3 within the same group. Comparisons between groups at each time-point were made by Chi-square test: ^a^ and ^b^ *p* < 0.001 between psychobiotics and placebo at T2 and T3. Parentheses represent the missing data extrapolated to the total number of patients.

**Table 4:** Psychobiotics improve stress status: the number of non-stressed patients (based on PSS-14 score, cut-off value of 28) progressively increased, whereas for those taking the placebo, it decreased.

	Psychobiotics			Placebo		
Patients	T1 *^,^ **	T2 *^, a^	Τ3 **^, b^	T1 ***	T2 ***	Τ3
Non-Stressed	90	105	97 (115)	96	89	35 (40)
Stressed	42	27	14 (17)	38	45	81 (94)
*Total number*	132	132	111 (132)	134	134	116 (134)

Comparisons within the same group were made by means of *McNemar test*: * *p* = 0.001 between T1 and T2 and T2 and T3 within the same group; ** *p* < 0.001 between Τ1 and T3 within the same group; *** *p* < 0.001 between T2 and T3 and T1 and T3 within the same group. Comparisons between groups at each time-point were made by Chi-square test: ^a^ *p* = 0.016 between psychobiotics and placebo at T2; ^b^ *p* < 0.001 between psychobiotics and placebo at T3. Parentheses represent the missing data extrapolated to the total number of patients.

## Data Availability

The data that support the findings of this study are available on request from the corresponding author K.K.

## Supplementary Materials

The following supporting information can be downloaded at: https://www.mdpi.com/article/10.3390/nu17050857/s1, Table S1: Baseline characteristics based on depression status within the same group.

## Abbreviations

The following abbreviations are used in this manuscript:

HDRS	Hamilton Depression Rating 17-item Scale
BDI-II	Beck’s Depression Inventory, 2nd Edition
GAD-7	General Anxiety Disorder-7
PSS-14	Perceived Stress Scale-14 Item
SCFA	Short-chain fatty acids
