# Supplementary material for: Psychobiotics Ameliorate Depression and Anxiety Status in Surgical Oncology Patients: Results from the ProDeCa Study
Source: Nutrients. 2025 Feb 28;17(5):857. doi: 10.3390/nu17050857 (PMC11901992; doi:10.3390/nu17050857)
Supplement: Supplementary file 1 [file nutrients-17-00857-s001.zip › nutrients-3466148-supplementary.pdf]

**Table S1:** Baseline Characteristics based on Depression status within the same group.

| | Psychobiotics | | p-value | Placebo | | p-value |
|---|---|---|---|---|---|---|
| **Number of patients** | 132 | | | 134 | | |
| Depression Status | **Non-Depressed (n=84)** | **Depressed (n=48)** | | **Non-Depressed (n=83)** | **Depressed (n=51)** | |
| Age (years-median-IQR) | 67.0 (15.0) | 71.0 (24.0) | 0.784 | 65.0 (17.0) | 65.5 (15.5) | 0.200 |
| Gender (M/F) | 50/34 | 38/10 | 0.021 | 55/28 | 35/16 | 0.777 |
| Weight (kg) | 76.5 (15.9) | 71.9 (12.6) | 0.088 | 73.2 (18.0) | 77.7 (17.8) | 0.211 |
| Height (cm) | 169.6 (9.1) | 172.9 (6.0) | 0.012 | 171.6 (8.7) | 168.4 (9.3) | 0.097 |
| BMI (Kg/m²) | 26.6 (4.9) | 24.1 (4.2) | 0.010 | 24.6 (4.8) | 27.2 (4.4) | 0.005 |
| Weight Loss (Yes/No) | 62/22 | 42/6 | 0.064 | 58/25 | 38/13 | 0.564 |
| Marity Status | | | | | | |
| Free | 14 | 8 | 0.670 | 14 | 8 | 0.049 |
| Married | 56 | 28 | | 57 | 33 | |
| Divorced/ Widow/er | 8/8 | 6/8 | | 6/6 | 0/10 | |
| Education | | | | | | |
| Basic | 18 | 12 | 0.435 | 22 | 13 | 0.449 |
| Secondary | 48 | 22 | | 34 | 26 | |
| University | 18 | 14 | | 27 | 12 | |
| Income | | | | | | |
| <10K Euros | 20 | 22 | 0.032 | 25 | 16 | 0.986 |
| 10-30K Euros | 60 | 24 | | 50 | 30 | |
| >30K Euros | 4 | 2 | | 8 | 5 | |
| Health Insurance | | | | | | |
| Public/None | 78/2 | 42/4 | 0.268 | 71/2 | 46/2 | 0.554 |
| Private | 2 | 0 | | 2 | 0 | |
| Both | 2 | 2 | | 8 | 3 | |
| Habits | | | | | | |
| Smoking (Yes/No) | 34/50 | 20/28 | 0.676 | 32/51 | 14/37 | 0.189 |
| Alcohol (Yes/No) | 8/76 | 8/40 | 0.226 | 19/64 | 7/44 | 0.193 |
| Sedatives (Yes/No) | 19/58 | 13/42 | 0.891 | 15/63 | 9/47 | 0.638 |
| Tumor Location | | | | | | |
| Gastric | 21 | 11 | | 20 | 13 | |
| Large Bowel | 41 | 24 | 0.907 | 42 | 22 | 0.485 |
| Rectum | 16 | 8 | | 17 | 10 | |
| Pancreas | 6 | 5 | | 4 | 6 | |
| Disease stage | | | | | | |
| Stage I | 9 | 7 | | 9 | 6 | |
| Stage II | 41 | 17 | 0.521 | 38 | 16 | 0.414 |
| Stage III | 27 | 19 | | 28 | 23 | |
| Stage IV | 7 | 5 | | 8 | 6 | |
| Charlson Comorbidity Index (median-IQR) | 4.5 (1.0) | 5.0 (1.5) | 0.117 | 5.0 (1.0) | 5.0 (1.0) | 0.653 |
| Side-effects of chemo-: | | | | | | |
| Diarrhea (Yes/No) | 20/64 | 14/34 | 0.498 | 26/60 | 11/37 | 0.364 |
| Vomiting (Yes/No) | 10/71 | 7/44 | 0.818 | 12/71 | 9/42 | 0.622 |
| Chemo- temporary dis-continuation (Yes/No) | 10/84 | 7/31 | 0.227 | 8/92 | 6/28 | 0.112 |
| Hair Loss (Yes/No) | 6/71 | 7/48 | 0.880 | 7/80 | 5/42 | 0.616 |

IQR: Interquartile Range; M: Male; F: Female.
